# “If only had I known”: a qualitative study investigating a treatment of patients with a hip fracture with short time stay in hospital

**DOI:** 10.1080/17482631.2017.1307061

**Published:** 2017-04-03

**Authors:** Charlotte M. Jensen, Anthony C Smith, Soren Overgaard, Uffe Kock Wiil, Jane Clemensen

**Affiliations:** ^a^ Department of Clinical Research, University of Southern Denmark, Odense, Denmark; ^b^ Department of Orthopaedic Surgery and Traumatology, Odense University Hospital, Odense, Denmark; ^c^ Centre for Innovative Medical Technology, University of Southern Denmark & Odense University Hospital, Brisbane, Denmark; ^d^ Centre for Online Health, University of Queensland, Australia; ^e^ The Maersk Mc-Kinney Moller Institute, University of Southern Denmark, Odense, Denmark

**Keywords:** Hip fracture, osteoporosis, self-care, patient empowerment, short time stay in hospital, phenomenology, reflective lifeworld research

## Abstract

Hip fractures are amongst the leading causes of admission to an orthopaedic ward. Systematized pathways with reduced admission time have become increasingly common as an essential tool for quality development and to improve efficiency in the hospital setting.  The aim of this study was to clarify if the patients feel empowered and able to perform self-care after short time stay in hospital (STSH) due to a hip fracture. The study used descriptive phenomenology to describe experiences of the pathway. Field studies were conducted in hospitals and in the patients' homes.  Interviews were performed with 10 patients recruited from two wards at a Danish University hospital, 4 family members and 15 health professionals from three hospitals.  The open attitude of reflective lifeworld research guided the analysis. The findings revealed that patients felt unprepared and insecure about their future, but also had a strong desire to be in charge of their own lives.  Of all the patients interviewed, none had any recollection of the information given to them by health professionals during their hospital admission. This study demonstrates that empowerment of patients with hip fractures is not adequately achieved in the pathway with STSH.

## Introduction

For decades, the general focus in orthopaedic surgery has been on improving the surgical treatment and rehabilitation of patients—this includes patients with proximal femoral (hip) fractures. At the same time, hospital admission time has shortened, prompting new challenges associated with early discharge. Although hip fracture treatments are commonly evaluated, there are gaps in our knowledge concerning the implications of pathways, which lead to reduced hospital admission time for the affected person.

Hip fractures are the most common cause of hospitalization in an orthopaedic ward (Palm, 2008; Parker & Johansen, 2006). Worldwide, the incremental societal burden of hip fractures is an important health problem—both for the patient in the form of functional decline and pain, for the families and for the society in a health economic perspective (Hansen, Mathiesen, Vestergaard, Ehlers, & Petersen, 2013; Metcalfe, 2008; Schiller et al., 2015).

Systematized fast-track programmes are an interdisciplinary, evidence-based multimodal concept aimed at improving peri-operative treatment in elective patient pathways (Husted, 2012; Walter, Smith, & Guillou, 2006). These systematized programmes have become increasingly common as an essential tool for quality development and to improve efficiency in the hospital setting (Kehlet & Dahl, 2003). Programmes focused on reducing length of stay in hospitals will most likely put more pressure on other health practitioners, who are expected to prepare patients for discharge from the hospital. Similarly, spending less time in hospital will also add to the demand on patients and their relatives.

The significance of both written and oral information in connection with hospitalization and discharge planning has been highlighted in studies within general contexts and specifically for older people who have sustained a hip fracture (Zidén, Scherman, & Wenestam, 2010). Presumably, this information or education of the patients aiming at empowering the patients is also challenged because of the short hospital admission period. How this is experienced by the patients has not been investigated before. Therefore, the aim of our study was to describe experiences of patients with a hip fracture and explore if the patients felt empowered and able to perform self-care in pathways with short time stay in hospital (STSH).

## Methods

### Study design

In this study the phenomenon of interest was “the hip fracture pathway with STSH”. The papers focus was on the patients’ perspective, but to gain a broader and richer description of the field of interest, we interviewed health professionals and conducted observational studies in three different hospitals in Denmark (an overview of health professionals’ profiles and place and duration of field observations are presented in Table 1). As the aim of the study was to describe *experiences* of the hip fracture pathway, a descriptive phenomenological approach was selected (Giorgi, 2009).

**Table 1.** Health professional interview profiles (*n* = 15).

Staff ID reference	Work environment	Organisation	Duration (min)	Field study
**Certificated healthcare worker (HP1)**	O2—orthopaedic ward	Odense University Hospital—Odense	50	Yes
**Nurse (HP2)**	O1—orthopaedic ward	Odense University hospital—Svendborg	60	Yes
**Physiotherapist (HP3)**	Rehabilitation unit	Odense University Hospital—Svendborg	60	No
**Charge-nurse (HP4) + research nurse (HP5)**	Geriatrics department	Aarhus University Hospital	60	Yes
**Charge-nurse (HP6) + nurse (HP7)**	Ortho-geriatrics	Kolding Hospital	70	Yes
**Phd-student (HP8)**	Ortho-geriatrics	Kolding Hospital	60	No
**Geriatric doctor, PhD (HP9)**	Geriatrics ward	Odense University Hospital—Odense	45	No
**Certificated healthcare worker (HP10) + physiotherapist (HP11)**	“Live and Home”—rehabilitation ward	Kragsbjergløkke, Odense Municipality	45	Yes
**Physiotherapist—leader (HP12)**	Municipality—Rehabilitation centre	Odense municipality	55	No
**Physiotherapist, leader (HP13)**	Municipality, rehabilitation	Odense municipality	60	No
**Physician—in charge of the patient pathway (HP14)**	Orthopaedics	Odense University Hospital—Odense	60	No
**Chief of staff—chief physician (HP15)**	Orthopaedics	Odense University Hospital—Odense + Svendborg	50	No

**Table 2.** Patient demographics at time of interview (*n* = 10).

Patient ID reference	Age (years/gender)	Living arrangements	Previous occupation
**P1**	67/F	Lives with husband, home with stairs, no professional carers	Assistant in clothing store
**P2Daughter present**	91/F	Widow, lives alone, sheltered housing, daughter nearby	Factory worker
**P3**	74/F	Widow, lives alone, home with stairs, children nearby	Leader of a kindergarten
**P4**	78/F	Lives with husband, 1-storey house, son nearby	Factory worker
**P5**	83/M	Lives alone, divorced, small apartment, daughter nearby	Cigar maker and “grinder”
**P6**	92/F	Lives alone, widow, sheltered apartment, brother nearby	Farmers wife and cleaning lady
**P7Husband present**	67/F	Lives with husband, house with stairs, no home service carers	Accountant
**P8**	73/F	Lives with husband, 3-storey house	Pharmacist
**P9Husband present**	81/F	Lives with husband, 3-storey house, daughter nearby	Nurse
**P10Wife present**	82/M	Lives with wife, home with stairs	Restaurant-manager

Phenomenology is a discipline that investigates people’s experiences to reveal what lies “hidden” in them (Giorgi, 2009).

In this study, the pathway is defined from the time the patients’ acquire the hip fracture until approximately 14 days after discharge from the hospital. The patient pathway varied in the three hospitals. Our findings concerning the patient perspective are derived from data achieved from the patient pathway with the shortest mean admission duration and with no statistically significant difference in re-admission rates, according to annual report from Danish Regions (2014), namely Odense University Hospital (OUH). Therefore, all patients involved in this study had been treated at OUH.

Participants gave their informed, written consent and approval was obtained from the Regional Health Service and University Research Ethics Committee and the Danish Data Agency (S-20110171; § 14, stk. 1; 2008-58-0035) (case approval no. 15/11860).

### Definition of key concepts

Patient empowerment is about strengthening and supporting patients’ own resources and capabilities to exercise self-care (Anderson & Funnell, 2010). According to the World Health Organisation (WHO), patient empowerment is “a process through which people gain greater control over decisions and actions affecting their health” (WHO, 1998, p. 25)

In our study, we measured empowerment by exploring these questions:

1. Did the patients know the available health services and the possibility of using these in relation to rehabilitation?
2. Were the patients able to cope with parts of or possibly the full rehabilitative treatment?
3. Were the patients able to take responsibility for their lives and did they seek to modify and maintain changes in life habits and so on in order to prevent new fractures?
4. Were the patients able to handle negative emotions either by themselves or in cooperation with their network or, for instance, other patients with similar problems?

We define self-care according to the Orem model of nursing (Orem, 2001) as “…practice of activities that individuals initiate and perform on their own behalf in maintaining life, health and well-being” (p. 117).

### Participants

A total of 29 informants were included in our study: 10 patients, 4 relatives and 15 health professionals (see Tables 1 and 2). The 10 patients were selected during the observation period according to the criteria of inclusion. If interested, the patients were contacted by the first author and they were informed briefly about the project. After their discharge they were contacted by phone and an interview was planned at a time of their convenience if the project still had their interest. None of the patients declined. Four of the patients had relatives present during the interview. We included patients who were discharged to their own homes and who had been independent prior to the hip fracture. That is, the patients had been able to walk around and perform everyday life without significant assistance from the municipality. Another criterion was that the hip fracture was a fragile fracture. That is, that the trauma mechanism of the hip-fracture was a result of falls from standing height—indicating osteoporosis. Patients were included from the two wards at OUH from June to December 2015 and interviews were conducted with patients with different working experience, different ages and gender. In all, 15 interviews were conducted with health professionals from different professions and healthcare sectors seeking a rich variation in data to broaden the description and the understanding of different nuances of the phenomenon.

### Data collection

We approached the phenomenon openly. Firstly, field observations (Spradley, 1980) were conducted within wards at the three different hospitals in Denmark, from May to November 2015. The focus was on the patients’ progress on the day and how the patients were prepared for life after discharge—that is, the rehabilitation after a hip fracture and a life with a possible chronic condition (i.e., osteoporosis).

Secondly, interviews were held with patients (P) and health professionals (HP), and field observations were conducted in patients’ homes. All primary interviews with the patients were conducted at their homes and as a conversation and the applied approach was focusing on specific lifeworld close descriptions (Dahlberg, Dahlberg, & Nyström, 2008; Kvale, 1996). Therefore, all the interviews started with the following invitation: “Tell me about your experiences while recovering from your broken hip.” The opportunity to seek clarification was used with prompts such as “Tell me more about it”, “What does this mean to you?” and “Can you clarify?” Interviews were carried out approximately 2 weeks after discharge, anticipating that patients would have recovered from the acute phase of their injury. All interviews were recorded and transcribed verbatim. A second interview was conducted by telephone 3–5 months after the primary interview. This comprised of a short follow-up interview to verify findings from primary interview, follow-up on the individuals’ current condition and whether they had anything to add since the initial interview.

Interviews with health professionals were made with semi-structured interview-guides compiled according to recommendations by Spradley (1979) and Kvale (1996). These interview-guides focused on three main themes: (1) the patient pathway from the HPs’ points of view; (2) their role in the pathway—how they involved and supported the patient; and (3) how they secured/supported the patient in connection with discharge. Field observations and interviews were done by the first author. Data, analysis and emerging descriptions were continuously discussed in circular processes within the group of co-researchers.

### Data analysis

The interviews with the patients, data from the field-observations and the interviews with health professionals were analysed together. In describing and understanding perspectives of a hip fracture and its consequences in everyday life we chose the phenomenological research approach, *Reflective Lifeworld Research*, to guide the data analysis (Dahlberg, 2010; Dahlberg et al., 2008). “Lifeworld” being the “world” each one of us lives in—the “background” of all experience. Understanding the patients’ perspective of acquiring a hip fracture and its consequences for their everyday life requires an ability to openly meet their lifeworlds—meaning the world “as lived” prior to reflective representation or analysis (Giorgi, 2009). Reflective lifeworld research illustrates the world as experienced prior to any theories devised to explain it (Dahlberg, 2010). Therefore, the analysis was based on principles from reflective lifeworld research (Dahlberg, 2010) in describing the essence and meaning structure of the phenomenon “hip fracture pathway with STHS”. Firstly, an overall impression of all the data was captured by several readings of data by the first author. Secondly, data were divided into meaningful topics. Thirdly, these topics were organized in groups and fourthly, these groups were organized into patterns that generated a general structure that was the essence of the phenomenon and its constituents. This discovering and creation of meanings was not made as a linear process but in circular processes back and forth in the material in the four phases. In our understanding of the phenomenon, attention was on bridling our evolving understanding of the phenomenon and its meaning. This being specifically important as the first author had a close knowledge of the orthopaedic field of care. “Bridling” means to reflect openly and respectfully upon the whole event when meanings come into being—not letting prior understanding overshadow new meanings and the phenomenon’s “otherness”(Dahlberg, 2010). Finally, we discussed the essence of the phenomenon according to the two key concepts empowerment and self-care.

In the findings, some of the major constituents of the essence of the phenomenon are presented. The four major constituents concerning the patient’s perspective include: (1) pre-conceived notions, (2) importance of autonomy, (3) “master in my own house” and (4) will and zest for life. These represent different aspects of the phenomenon and put together they comprise the phenomenon as a whole. The results are based on all interviews but individual statements illustrate and highlight the descriptions.

## Findings

A hip fracture is a serious injury, with potential complications that can be life-threatening. Systematized guidelines including rapid mobilization are introduced as a tool for quality development and improvement of efficiency in pathways with reduced hospital admission time. The patients in our study were hospitalized for an average duration of 5.6 days. In order to implement pathways with reduced admission time, the health professionals’ tasks require standardized preparation and stringency and conformity. This standardized way to treat patients may compromise the patients’ wish to be involved and treated as individuals. The focus in the hospital is recovery and rehabilitation, whilst the focus for the patient is how this injury will change the way in which they live.

Acquiring a hip fracture severely interferes with an individual’s life and personal concern for the consequences of the hip fracture on life after hospitalisation. In the standardized and systematized pathway with STSH, health professionals continuously seek to inform patients of what to expect during and after hospitalization but patients cannot retain all the information. Likewise, they cannot always participate in specialised interventions such as fall-prevention programmes and dual-energy X-ray absorptiometry (DXA) scanning, a means of measuring bone mineral density. Lack of knowledge and insecurity towards future expectations is therefore a significant issue for the patients.

Patients can be uncertain how to best manage their life after hospital treatment. On one hand they have a wish to postpone the unmanageable situation, but on the other hand they wish to be able to take charge of the situation as they have done prior to the hip fracture. Interactions with health professionals tend to focus on the physical problem (the hip fracture) and the rehabilitation, while their frail existence may end up being perceived as less important. The healthcare professionals provide recommendations that may lead the patients to passively take in advice and information. When at home they choose their own strategies for their continued self-care and rehabilitation and the understanding of advice and information given by the health professionals on a more profound level is not reached.

The inability to comprehend a large amount of information within a relatively brief period of time may also lead to the feeling of insecurity and the feeling of not being in charge of the situation. These factors may reduce the success required for patients to modify and maintain positive changes in activity and healthy living, in order to prevent new fractures. The patients have a wish to regain their physical ability but they do not feel empowered to do so.

### Patient perspectives of the pathway

#### Preconceived notions

Patients were able to explain their general understanding of their injury and prognosis. Comments from patients included  existential concerns as:

A hip fracture is a serious injury, with complications that can be life-threatening.

A hip fracture can reduce your future independence and sometimes even shorten your life.

About half of people who have a hip fracture are not able to regain their ability to live independently.

These preconceived notions seemed to place patients in a crisis-situation during hospitalization and days after discharge from hospital. The following statements illustrate some concerns:

I thought, oh no, now I have become one of them. (P9)

I was just lying in the bed [at the hospital] the first two days…with the bedcover over my head…crying and thinking: oh am I now going to a nursing home…and everybody will forget about me. (P4)

…this is not the way I had thought of to end my days. (P10).

Having the “disease” (the hip fracture), as many called it, and the need for hospitalization was an exhausting experience for all of the patients. The operation and hospitalization was physically exhausting. The concerns of being a patient with a hip fracture and the potential long-term complications were mentally exhausting. With limited knowledge, patients felt insecure and could not grasp what their situation at home would be like after discharge.

#### Importance of autonomy

When telling their story only one of the patients and her daughter recalled their time at the hospital *before* the operation. The patients all described the pathway in the factual details. They did not question the structure of the pathway.

None of the patients or their relatives expressed having been involved during processes of hospitalization. They had received information like “we usually discharge patients like you after about five days” and “now you have to get out of bed”. Medicine was placed on their table and medicine lists and discharge letters were delivered at the time of discharge. They had a perception that the list and letter was something they had to give to the community nurse—they did not feel it was targeted at them. Most of the patients accepted that this apparently was the way it should be and they did not question this although they expressed a desire to be involved in the discharge plans. One expressed the humiliation of not feeling like she was being treated as a human being:

…even if you are old, they ought to see you as a human being. It is not our fault that we have become old and got some flaws. There should be room for us anyway… (P3)

They mainly found it understandable that the HP’s had a routine way of providing patient care because they had so much to do:

…one cannot say that they are lazy…they run around like busy bees. (P3)

…we see many different people…but that is ok…they seem to work on the same recipe or scheme… (P4)

Lack of knowledge in connection with future expectations was a significant issue. Almost all of the patients stated insecurity in connection with discharge.

#### Master in my own house

A common theme expressed by patients was the importance of being in charge of their own lives and to be “masters in their own house”. They expressed a desire to know “what to expect”. They all expressed their surprise at how quickly their physical capacity progressed at home:

If only had I known…I would not have been so devastated about discharge. (P10)

The first week after I came home I actually got better every day. (P1)

The majority of patients also expressed that shortly after they returned to their homes they renounced the home based services provided by the municipality. This was done because the municipality could not provide them with service or assistance in accordance with their needs and they had no desire to conform to what the municipality could offer.

They felt that health professionals had taken charge of situations that they themselves previously had been in charge of. This was both in the hospital setting and after discharge:

…I felt completely left out…and I couldn’t grasp how I was going to be able to take my part in that…and it was stressful because I do not want to pull tough toll [making his wife have all the tasks and the responsibility] on my wife, of course not” (P10)

#### Will and zest for life

Most of the patients had a pragmatic approach to the future. Patients were accepting of their role in rehabilitation after the hip fracture and understood that this would be challenging:

If you are the son of God, then you take care of yourself. (P2)

I think it will be hard…but we will just have to take one day at a time…now the damage is done, so it will just have the time needed to be ok again. (P5)

The patients expressed no knowledge of how to live their lives henceforth in accordance with prevention of new falls and fractures. When asked about osteoporosis they were dismissive. They said that they were not receptive to getting more facts about this other than just wanting to recover from “the disease”.

We found that these patients coming from and returning to their own homes had a zest for life that enhanced opportunities for rehabilitation. Nevertheless, all patients declared that praise and knowledge of the normal pathway would promote empowerment:

Now…I am very stubborn and I *know* that I will get through this…but I can very well imagine other elderly people sitting there…and they have no will to fight…because they need to be praised to carry on and who should do that if you are on your own? (P2)

According to the national standard hip fracture programme, a hip fracture caused by a low-energy trauma is associated with osteoporosis (Danish Regions, 2014; Biostatistik, 2016). According to the local clinical guidelines, patients older than 65 years were prescribed calcium tablets and offered a DXA-scan post-discharge (Lauritzen, 2016). In our study, we found the majority of patients did not understand the reason for being treated with calcium. Most patients and their relatives presumed this treatment had something to do with the healing of the bones. Patients planned to stop taking the calcium tablets once they felt their leg or hip was OK. None of the patients liked taking the tablets mainly due to side-effects such as gastrointestinal discomfort. One patient explained that having to take tablets made her feel more like a sick person than just a person with a fracture. This patient did not want to think about having osteoporosis:

It is bad enough that I have to recover from having the hip fracture. If I constantly have to think about that I might have osteoporosis then I would really feel sick. I cannot concentrate on that now. (P6)

Only one of the patients had the full perception of what the different offers of preventive control visits at the hospital were and what they meant. None of the patients wanted to attend a fall-prevention programme. They all had the notion that their fall was explainable and accidental. Eight patients had agreed to a DXA-scan but when they realized that the scan was not a way to check healing of the hip fracture, only five had the scan. They all felt that the time of the scan was very inconvenient. One thought that it was a total waste of time:

Why scan me? I am more than 80 years old. Of course my bones are not as strong as when I was young. That is logical. And so what? (P10)

### Self-care and empowerment

The staff at the hospital stressed the positive effects of having systematized guidelines aiming at reduced hospitalization: it diminishes the risks of patients in delirium, and with rapid mobilization patients are not seen as sick but more as having been treated for a physical defect. The challenges in effecting this were seen in what the municipality were able to offer in order to follow through on the rehabilitation. The care pathway was made according to these systematized, local clinical guidelines reflecting the National Clinical Guidelines aiming at STHS and the staff claimed that this required preparation, stringency and conformity.

#### Preparing for discharge

Our field studies showed that the staff sought ways to support patients’ self-care. They encouraged patients to do as many everyday life activities as possible to restore their regular daily life and muscular capacity. They encouraged the patients to do as much as possible of their personal hygiene and they encouraged the patients to get out of bed and to walk to the toilet instead of getting a bedpan and so on. Almost all of the patients, however, had no recollection of this in rehabilitative terms and thought of rehabilitation only in terms of the physiotherapy training provided in either their own homes or at the training centre in the municipality.

There was consensus amongst staff that patients should be individually informed about the pathway and what they (the patients) should expect after discharge. Staff reported that they assessed the needs of the patient to determine if and what written information should be provided.

However, there was also an awareness of the need to customise the information more towards the specific needs of each individual patient:

It may well be that we as a staff just communicate with each other and we set a frame for how the process should be…it may well be that we do not get our knowledge disseminated to the patients. (HP12)

#### Cross sectional collaboration

The different health professions had no exact knowledge of what to expect from the other professions. The staff in the hospital setting thought that the reason why the patients were afraid of discharge was because they were afraid of falling. Likewise, they were of the opinion that the municipality were not able to provide immediate rehabilitation and support the patients in their homes in a sufficient and satisfying way.

Representatives from the municipal rehabilitation, on the other hand, were aware of the fact that not much of the information given to the patients at the hospital was memorized:

Because individuals can only take this much in when you are in a stressful situation. It is our obligation to follow up on this information. The pathways are now so accelerated that patients cannot absorb more. (HP15)

Therapists from the municipality expressed that they had brief contact with the patients and were cognisant of the challenges this created:

They cannot start with half an hour of training if the pathway is so accelerated that that they have not yet come home in their own shoes mentally…we are only there for a short time but citizens live their lives the entire day…I am only in here for half an hour twice a week and then what happens? (HP15)

## Discussion

The findings reported in this study are consistent with other published studies that illustrate the challenges of providing appropriate care for patients with an osteoporotic hip fracture (Beer & Giles, 2005; Griffiths et al., 2015). The accelerated treatment plan has become a commonly used tool for the standardisation of care and for the preparation of patients for early discharge from hospital. However, we found some important shortcomings related to patient support during hospitalization and rehabilitation. This correlates with findings from The Danish Institute for Health Services about future care in healthcare. They found that fast-track treatments require better communication and greater involvement of patients in the care planning process. In relation to this challenge, a study involving another surgical patient cohort identified that patients can experience an asymmetry between their degree of influence and a power-related asymmetry manifested by healthcare professionals within the practice of fast-track programmes (Norlyk & Harder, 2009). This is reflected in our study, where the patients experience a lack of involvement and influence in for example the discharge plans.

Very importantly, we found that patients expressed a desire to be treated as autonomous individuals and to be provided with the necessary knowledge to enable them to improve and maintain their health. Empowerment is a concept that focuses on the processes through which people can improve their ability to develop, control and manage their resources (Anderson & Funnell, 2010). A way of doing this could be providing people with the necessary knowledge. The health professionals were aware of providing the patients with both written and oral information. Nevertheless, our study showed that this way of empowerment of patients with hip fractures was not achieved during a STSH. Establishing this was challenged by the fact that patients’ pre-existing concerns masked their state of mind during hospitalization. The general need for information and the feeling of lack of information was a significant factor for the individuals in our study. The lack of information added to the feeling of insecurity at the time of discharge from hospital. They did not know about the available health services or the possibility of using these in relation to rehabilitation. Other studies on quality of life in orthopaedic patients have reported similar findings (Archibald, 2003; Malin Malmgren, Eva Törnvall, & Inger Jansson, 2014), where participants felt insignificant and ignored. The concept of self-care is a many-layered one (Godfrey et al., 2011). Our findings support the definition of self-care that individuals primarily need to have a feeling of being in charge of their own lives to be able to perform self-care. In addition, individuals need to feel that they can make informed choices. Orem’s model (Orem, 2001) proposes that nursing should be especially concerned with the patient’s need to move continuously towards responsible action in self-care in order to sustain life and health or to recover from disease or injury. This means that patients have to have access to appropriate information at the right time to ensure that this helps them adapt to their treatment and recovery. This echoes findings in a study on experiences of living with a new osteoporosis diagnosis where improved support to gain understanding of their diagnosis is suggested (Hansen, Konradsen, Abrahamsen, & Pedersen, 2014). In our study all patients had a strong will to regain control of their lives. However, they felt a need for information or knowledge concerning management of their future living.

Living longer is often considered an indicator of success in the modern world, but ageing does have its limitations—such as frailty. Several international studies have shown that fall-prevention programmes amongst elderly people are successful (El-Khoury, Cassou, Charles, & Dargent-Molina, 2013; Gillespie et al., 2009; Gschwind et al., 2013). Likewise, a recent study (van Velsen et al., 2015) has been made on the successfully development of a community-supported technology supporting the different phases of screening and offering training services. In Denmark, fall-prevention programmes are routinely offered to patients at risk of injury. Our study revealed that patients had no recollection of this, nor did they think that fall-prevention programmes were necessary. This corresponds with national (Evron & Lene, 2016) and international studies (Berlin Hallrup, Albertsson, Bengtsson Tops, Dahlberg, & Grahn, 2009) and a report from the Danish Health Institute (Sundhedsinstitut, 2011) acknowledging that older people do not necessarily see themselves as “such an old person who is susceptible to falls”. Therefore, they are less likely to participate in these general preventative programmes.

A systematic review (Marsh et al., 2011) shows that osteoporosis education and falls prevention reduces the incidence of fractures and the potential costs of treatment. In our study we found that this secondary prevention was included in the pathway: patients were offered both examinations and they were informed of the importance of this during hospitalization—both orally and in a written pamphlet. However, this form of information or education of patients was not obvious for the patients. Patients did not take this information into consideration during hospitalization and they had limited or no recollection of this after discharge. That is, they had no knowledge of how to seek to modify or make changes in life habits in order to prevent new fractures. This lack of knowledge inducing motivation to follow certain recommendations (such as the calcium treatment) corresponds with the findings in a cross-national study of use of osteoporosis medications after hospitalization for hip fracture (Kim et al., 2015) and challenges prevention of secondary fractures. In a lifeworld perspective, a study (Berglund, 2014) shows that the carers should proceed from the patient’s need for learning and not from ready made programmes. Challenges in supporting the patients’ ability to remember the information given to them by health professionals is stressed in other studies. This concerns both the pre-operative information of the elective patient pathways (Aasa, Hovbäck, & Berterö, 2013) and is concluded in a literature review on patient comprehension of discharge instructions from the emergency department (Alberti & Nannini, 2013). Further dialogue with existing literature on these findings would be interesting in future studies.

In our study we have shown that acquiring a hip fracture severely interferes with an individual’s life. Individuals worry about the consequences of the hip fracture on their future existence. From the patients’ perspective, a substantial amount of motivation and self-preservation were needed to do the rehabilitation and regain physical ability. Patients were very interested in acquiring knowledge to support them during rehabilitation and to help them reduce their risk of subsequent injury. The importance of responding to patient needs and creating a sense of empowerment is also highlighted in a Canadian study (Schiller et al., 2015), where patients were interviewed up to 3 years post hip fracture. In our study, we showed that this sense of empowerment was not achieved from the patients’ perspective.

## Conclusion

This study demonstrates that patients recovering from hip fractures, and who have been independent prior to the hip fracture incident, have a strong desire to be in charge of their own lives and to remain autonomous. We found that the empowerment of patients with hip fractures was not adequately achieved in the existing pathways associated with STSH. Patients seemed to accept these pathways, but acquiring a hip fracture and recovering from this type of injury is a traumatic experience—not just physiologically but also from a psycho-social perspective. Further studies that focus on improved methods of communicating health information and encouraging patient empowerment are required for patient autonomy and self-care, especially in cases with STSH.
